# Dual-Mode Embedded Impulse-Radio Ultra-Wideband Radar System for Biomedical Applications

**DOI:** 10.3390/s24175555

**Published:** 2024-08-28

**Authors:** Wei-Ping Hung, Chia-Hung Chang

**Affiliations:** 1 Department of Computer Science and Information Engineering, National United University, Miaoli 306301, Taiwan; 2 Department of Electrical Engineering, National Yunlin University of Science and Technology, Yunlin County 64002, Taiwan

**Keywords:** DC offset, embedded system, impulse radio radar, material sensing, ultra-wideband (UWB), vital sign

## Abstract

This paper presents a real-time and non-contact dual-mode embedded impulse-radio (IR) ultra-wideband (UWB) radar system designed for microwave imaging and vital sign applications. The system is fully customized and composed of three main components, an RF front-end transmission block, an analog signal processing (ASP) block, and a digital processing block, which are integrated in an embedded system. The ASP block enables dual-path receiving for image construction and vital sign detection, while the digital part deals with the inverse scattering and direct current (DC) offset issues. The self-calibration technique is also incorporated into the algorithm to adjust the DC level of each antenna for DC offset compensation. The experimental results demonstrate that the IR-UWB radar, based on the proposed algorithm, successfully detected the 2D image profile of the object as confirmed by numerical derivation. In addition, the radar can wirelessly monitor vital sign behavior such as respiration and heartbeat information.

## 1. Introduction

Recently, wireless and real-time detection using Doppler radar for healthcare applications has gained significant attention. Biomedical or physiological information such as respiration and heartbeat can be captured by microwave sensing to achieve long-term monitoring in hospitals [1]. An impulse-radio (IR) ultra-wideband (UWB) radar with the characteristics of continuous monitoring and low-power consumption is considered a great candidate to supersede conventional medical electrodes [2]. Several studies on the IR-UWB radars for vital sign detection have been reported in [3,4]. In addition to vital sign application, microwave imaging is widely used in non-invasive inspections, remote sensing, or biomedical images for further diagnostics and treatments. The emitted microwave energy is imposed on a dielectric material, accompanied by the transmitted, reflected, and scattering waves. The probing signal experiences a static object, and the corresponding energy is then reflected back to the radar sensor for the following signal processing. However, material sensing with microwave imaging suffers from inverse scattering issues [5,6], which can degrade the quality of the received signal and image. In addition, the direct current (DC) offset caused by the reflected waves or clutter signals is another crucial issue that can degrade receiving sensitivity not only in microwave imaging but also in vital sign monitoring. Various approaches, such as quadrature radar architecture, DC clutter canceller, DC compensation circuit, and servo feedback loop [7,8,9,10,11,12], have been developed to overcome the DC offset for vital sign applications. Nevertheless, the DC offset with time-variant behavior deviates from different types of material objects. Accordingly, a digital-assisted algorithm with a self-calibration technique is proposed to calibrate the received DC level. This study develops an integrated IR-UWB radar sensor that can simultaneously perform biomedical imaging and vital sign monitoring. The main contributions are summarized as follows:

(1) Instead of using a commercial radar module that serves a single purpose in the previous works, a customized front-end circuit featuring a dual-channel analog signal processing circuit (ASP) has been developed for detecting object contours and monitoring life activities. This compact and portable radar device is integrated into an IR-UWB system for both applications.

(2) An inverse scattering equation with the iterated τ is developed to overcome the nonlinear issue for a digital imaging algorithm, which is implemented in an embedded system. A self-calibration mechanism for the DC offset issue is also incorporated into the algorithm to achieve real-time and adaptive calibration. This concept has the capability to identify material properties by using statistical or artificial intelligence techniques.

## 2. Design of the Embedded IR-UWB System

**Figure 1:**
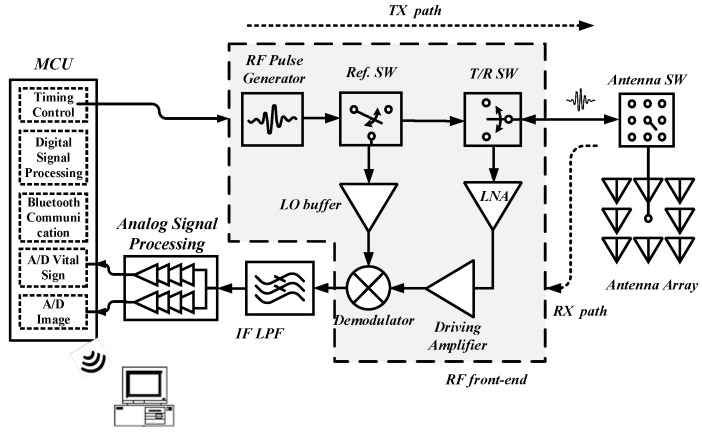
System architecture of the IR-UWB radar for hybrid biomedical applications.

The system architecture of the proposed IR-UWB radar system with dual applications is shown in Figure 1. The radar module comprises an RF front-end block, a switching antenna array, a dual-path ASP, and the data processing block. The RF front-end circuit uses a monostatic antenna system to transmit a periodic sequence of nanosecond pulses at a pulse repetition frequency (PRF) and subsequently receives the reflected waves. These waves are demodulated to baseband for additional signal processing. The transmission and reception modes are determined by the T/R switch and Ref. switches, which are controlled by the microcontroller (MCU) [10]. In addition, the antenna is a crucial component of the radar, as it is responsible for detecting the signal transmission and echo reception. An antenna array with eight sub-array antenna units forms a square shape to increase the effective sensing area for image detection. Further details on the design concept and implementation are provided in the following sections.

### 2.1. Hardware Implementation

The IR-UWB radar detects the object or patient’s chest by correlating the phase delay between the probing and reflected pulses. A fixed sensing distance of *R_o_* between the target and the radiated antenna can be obtained by *R_o_ =* (*C*∙*t_d_*)/2, where *C* is the speed of the electromagnetic (EM) wave, and *t_d_* is the delay time of propagation. To achieve this, a series of probing pulses are provided by the RF pulse generator [13] and coupled to the antenna array. In this work, a probing RF pulse sequence with a resonant frequency of 5.5 GHz, a pulse repetition frequency of 2 MHz, and a radio pulse width of 3 ns is designed. The reflected waves carry the EM energy with image and physiological information back to the radar module, which are then down-converted into intermediate frequency (IF) signals for analog and digital processing. Assuming that a sensing distance of *R_o_* between the target and the radiated antenna is fixed, the captured signal *Z*(*t*) can be characterized as follows:

(1) $$Z\left(t\right)=E_{eff}\cos\left(\phi \left(t\right)+\Delta \phi \right),$$

where *E_eff_* is an effective amplitude of the receiving signal; the Δ*ϕ* represents a fixed phase delay or spreading time between the radiated waves to the target and back to the antenna; and time variant *ϕ*(*t*) is an instantaneous phase value, which is determined by the sensing object or life activities behavior. It is observed that Z(t) is expressed as a demodulated signal with the target information of *E_eff_*, *ϕ*(*t*), and Δ*ϕ*.

**Figure 2:**
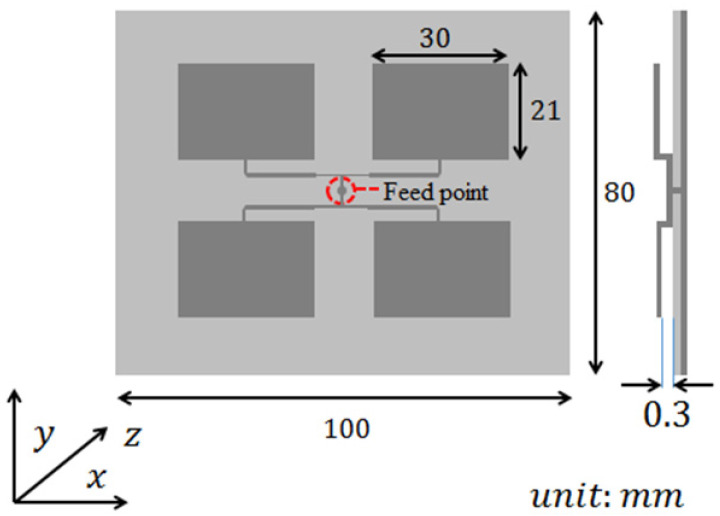
Structure of antenna unit.

Accordingly, from the perspective of microwave imaging, the antenna’s performance is critical in enhancing *E_eff_* and thereby determining the sensing quality. Herein, a monostatic antenna system comprising eight sub-array antenna units controlled by an antenna switch is adopted to achieve larger sensing coverage for object contour. Additionally, considering the antenna design, the conventional resonant antenna is unsuitable for IR-UWB radar detection due to the short-time nanosecond pulse, which might produce ringing phenomena and lead to severe waveform distortion [14]. Thus, a square patch antenna with a terminated load is adopted at the sensor output for nano-pulse radar application [15,16]. Figure 2 illustrates the prototype of the antenna unit design with an input impedance matched network, radiated at a wide frequency band from 5.1 GHz to 5.7 GHz to avoid process variation. The feeding point is located at the center of the module for measurement convenience. Subsequently, this prototype is used as a sub-array unit for planning eight switching antennas in a square shape. A one-to-eight power divider is utilized to equally distribute the transmitting power to each antenna unit.

**Figure 3:**
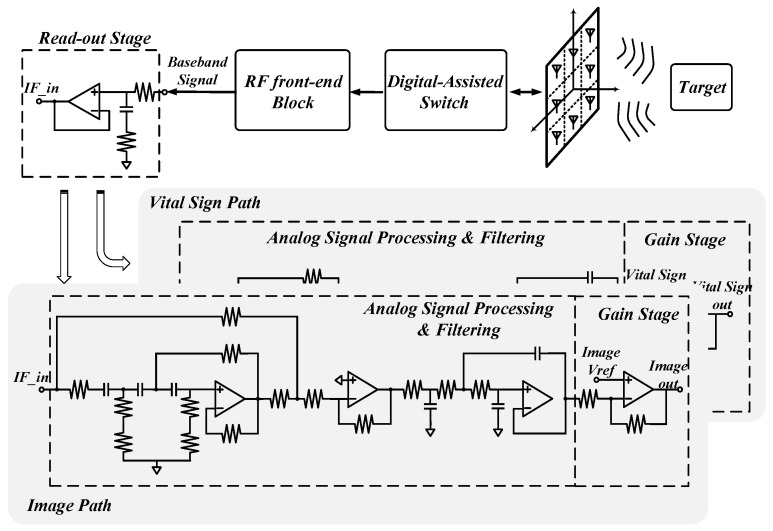
Dual-channel ASP for microwave imaging and vital sign data.

The captured signal, *Z*(*t*), is first demodulated to the IF and then passed through the analog circuits consisting of dual channels for image and vital sign applications, as shown in Figure 3. The processing block for vital sign path is composed of a read-out stage, a low-pass filter (LPF) with Chebyshev behavior, a high-pass filter (HPF), and a gain stage. The ASP block with a bandpass frequency response is designed to reject out-of-band interference [10,17,18]. On the other hand, the image channel bypasses the HPF without DC rejection. The DC components remain for recording each DC value from eight antenna units. These DC values are then compensated by the proposed self-calibration algorithm to avoid image distortion. This allows for the real-time sensing of object profile or vital sign information, which can be wirelessly transmitted via the Bluetooth (BT) interface. The data can then be visualized using the graphical programming language, LabVIEW.

### 2.2. Data Processing Block and Algorithms

**Figure 4:**
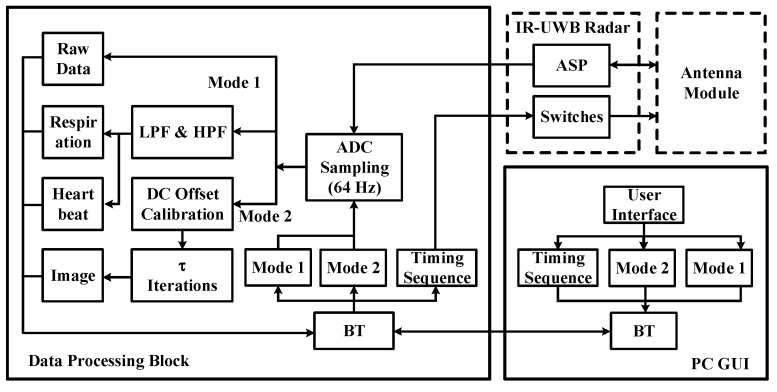
Dual-channel ASP for microwave imaging and physiological data.

The block diagram of the data processing module and graphical user interface (GUI) is illustrated in Figure 4. A BT module is adopted to communicate with others for functional selection and timing sequence of all controlling switches. The system integrates two modes for vital sign acquisition and imaging reconstruction. The sensing data from the IR-UWB radar are digitized at a speed of 64 Hz/s with a 16-bit resolution analog-to digital converter in all modes. Mode 1 serves the purpose of transmitting the captured raw data of the vital signs from the IR-UWB sensor and its corresponding processed signals via digital filter with the HPF/LPF difference equations. The main algorithm of the vital sign is similar to our previous studies [19,20], except for the power spectral density and peak detection. Instead of searching the peak value of the estimated respiration and heartbeat rates, these signals are directly transmitted to a personal computer (PC), and then the real-time vital signs are observed via the BT module.

Mode 2 is responsible for image reconstruction. The sampled data from the radar sensor are fed into a processing sequence, including DC offset calibration and estimated dielectric constant iterations, τ. The DC level compensation from each antenna unit is initially considered to avoid signal distortion. The procedure for the proposed self-calibration algorithm in the first step is to record the DC values of the eight sub-array antenna units, which are switched in turn to detect a 2D object profile [21,22]. Then, a dataset of DC values can be obtained by n times antenna scanning to evaluate the background information, where n can be determined by the user interface. The mean value is adopted to compensate the required DC level for antenna array. Furthermore, an estimated τ from the sensing object is required to depict object contour. Accordingly, the microwave imaging algorithm is developed to deal with the inverse scattering issue. For the sake of brevity, the complicated integral equations and microwave theory are omitted. The basic concept and the corresponding procedure are listed as follows. The electric scattering field is initially established. An inhomogeneous object is assumed to have permeability of $\mu_{0}$ and permittivity of $\varepsilon_{0}$ in free space. The total electric field E is the sum of the incident electric field $E^{i}$ and the scattering field $E^{s}$. The scattering field in the z-direction can be expressed as follows:

(2) $$E_{z}^{s}(\vec{r})=\int_{s}G(\vec{r},{\vec{r}}^{\prime })k_{0}^{2}(\varepsilon_{r}({\vec{r}}^{\prime })-1)E_{z}({\vec{r}}^{\prime })ds^{\prime },$$

where $G\left(\vec{r},{\vec{r}}^{\prime }\right)$ is the 2D Green function, and $k_{0}$ is a wave factor in the free space. The relative permittivity $\varepsilon_{r}$ and the total electric field $E_{z}$ can be expressed as follows:

(3) $$\varepsilon_{r}(x^{\prime },y^{\prime })=\sum_{n=1}^{N}\varepsilon_{rn}P_{n}\left(x^{\prime },y^{\prime }\right),$$

(4) $$E_{z}(x^{\prime },y^{\prime })=\sum_{n=1}^{N}E_{zn}P_{n}(x^{\prime },y^{\prime }),$$

where $\varepsilon_{rn}$ and $E_{zn}$ are the permittivity and the total electric field with the nth slicing area in the z-direction, respectively; $P_{n}\left(x^{\prime },y^{\prime }\right)$ is the basis function. Similarly, the scattering field and incident field can be expressed as follows:

(5) $$E_{z}^{s}(x^{\prime },y^{\prime })=\sum_{n=1}^{N}E_{zn}^{s}P_{n}(x^{\prime },y^{\prime }),$$

(6) $$E_{z}^{i}(x^{\prime },y^{\prime })=\sum_{n=1}^{N}E_{zn}^{i}P_{n}(x^{\prime },y^{\prime }),$$

Based on Equations (3)–(6) and the corresponding electric field integral equation, the scattering field can be further derived in a matrix form as follows:

(7) $$\left[E_{z}^{s}\right]=-[G_{2}][\tau_{z}]{\left([G_{1}][\tau_{z}]-[I]\right)}^{-1}(E_{z}^{i}),$$

where ${\left(\tau_{z}\right)}_{n}=\varepsilon_{rn}(x,y)-1$.

In addition, in order to simplify the integration operation of $\left(G_{1}\right)$ and $\left(G_{2}\right)$, each small area may be replaced with a circle of equal area. Here, $\left(G_{1}\right)$ and $\left(G_{2}\right)$ is

(8) $$\begin{array}{ll} {\left(G_{1}\right)}_{mn} & =-\frac{j\pi k_{0}a_{n}}{2}J_{1}(k_{0}a_{n})H_{0}^{\left(2\right)}(k_{0}\rho_{mn})\;m\neq n\\ & =-\frac{j}{2}[\pi k_{0}a_{n}H_{1}^{\left(2\right)}(k_{0}a_{n})-2j]\;m=n \end{array}$$

(9) $${\left(G_{2}\right)}_{mn}=-\frac{j\pi k_{0}a_{n}}{2}J_{1}(k_{0}a_{n})H_{0}^{\left(2\right)}(k_{0}\rho_{mn})$$

Here, $J_{1}$ is the first-order Bessel Function of the first kind, $a_{n}$ is the equivalent circle radius of the n-th small region, $\left(x_{n},x_{y}\right)$ is the center position of the n-th small region, and

(10) $$\rho_{mn}=\sqrt{{\left(x_{m}-x_{n}\right)}^{2}+{\left(y_{m}-y_{n}\right)}^{2}}$$

Thus, to establish the inverse scattering formula, the nonlinear equation of (7) can be solved by an iteration technique to obtain the dielectric constant $\left[\tau_{z}\right]$, which can be further expressed as

(11) $$[G_{2}]{\left([I]-[\tau ][G_{1}]\right)}^{-1}[\delta \tau ]{\left([I]-[G_{1}][\tau ]\right)}^{-1}[E^{i}]=[\delta E^{s}],$$

It is observed that the factors of [δτ] and $\left[\delta E^{s}\right]$ are linear dependent, where [δτ] is a diagonal matrix. Equation (11) can be expressed as a linear relationship, as shown in Equation (12)

(12) $$\left[P_{\delta }][x_{\delta }]=[Q_{\delta }\right]$$

where $\left[P_{\delta }\right]$ is related to $[G_{2}]{\left([I]-[\tau ][G_{1}]\right)}^{-1}$ and ${\left([I]-[G_{1}][\tau ]\right)}^{-1}[E^{i}]$. $\left[Q_{\delta }\right]$ is related to $\left[\delta E^{s}\right]$.

Consequently, the initial and actual dielectric constants are assumed to be $\left[\tau_{initial}\right]$ and $\left[\tau_{actual}\right]$, respectively. In the first iteration, an estimated scattered field $\left[{\hat{E}}^{s}\right]$ can be calculated by substituting $\left[\tau_{initial}\right]$ into Equation (7). $\left[\delta E^{s}\right]$ is determined by the difference between $\left[{\hat{E}}^{s}\right]$ and $\left[E^{s}\right]$. Herein, [δτ] can be obtained from Equation (8), thereby enabling an updated dielectric constant for the next iteration.

Consequently, the vital sign information and image profile can be obtained by the proposed algorithm, where the corresponding mode selection is determined by the user end. It is noteworthy that only a single antenna is required for vital sign detection because the sensing target focuses on the subject’s chest movement. The continuous time domain and frequency spectral of vital signs can be observed on GUI.

## 3. Experimental Results

**Figure 5:**
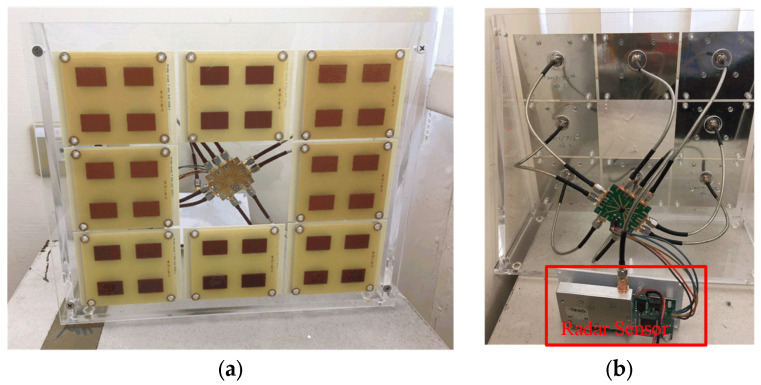
(**a**) Antenna array module with eight 2 × 2 square patch antenna units and (**b**) backside view of the implemented radar sensor.

Figure 5 displays photographs of the implemented IR-UWB radar sensor, a square-shaped antenna array, and a one-to-eight power divider/combiner, all of which are fabricated on the standard four-layer FR-4 printed circuit board. The physical sizes of the radar module and a 2 × 2 antenna unit are 9.9 × 5.7 cm^2^ and 8 × 10 cm^2^, respectively. A housing package is implemented in the RF front-end block to prevent radiation and coupling issues. The use of eight antenna units with digital switching control helps increase the overall sensing area. Table 1 provides the specifications of the main components used in this customized radar system.

**Figure 6:**
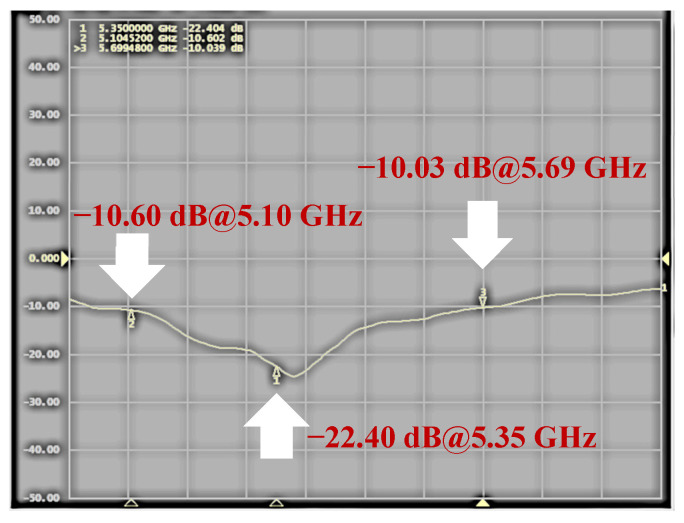
Measured return loss of the antenna unit.

**Figure 7:**
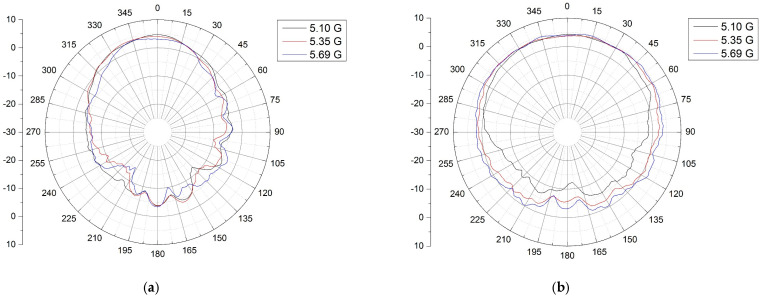
Measured (**a**) E-plane and (**b**) H-plane of the antenna unit.

The RF probing pulses are radiated and received by the transmitting/receiving antenna unit. The captured waves carry the desired information based on the Doppler effect. The antenna unit’s performance was evaluated by measuring the return loss, which was found to be lower than −10 dB within the frequency range of 5.10–5.69 GHz, indicating a wideband characteristic, as shown in Figure 6. The radiation patterns of the antenna unit were also measured on both the E- and H-planes, as depicted in Figure 7a and Figure 7b, respectively. The gain patterns at frequencies of 5.1 GHz, 5.35 GHz, and 5.69 GHz exhibited uniformity and a wide directional behavior across the entire frequency band. Thus, eight individual antennas forming an antenna set were adopted for the microwave image, while one of the antenna units was employed for vital sign monitoring.

**Figure 8:**
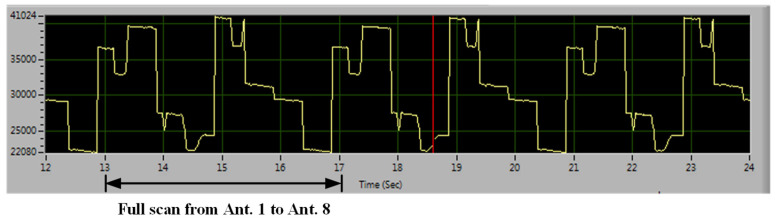
Recorded real-time signal with a periodical full scan antenna.

**Figure 9:**
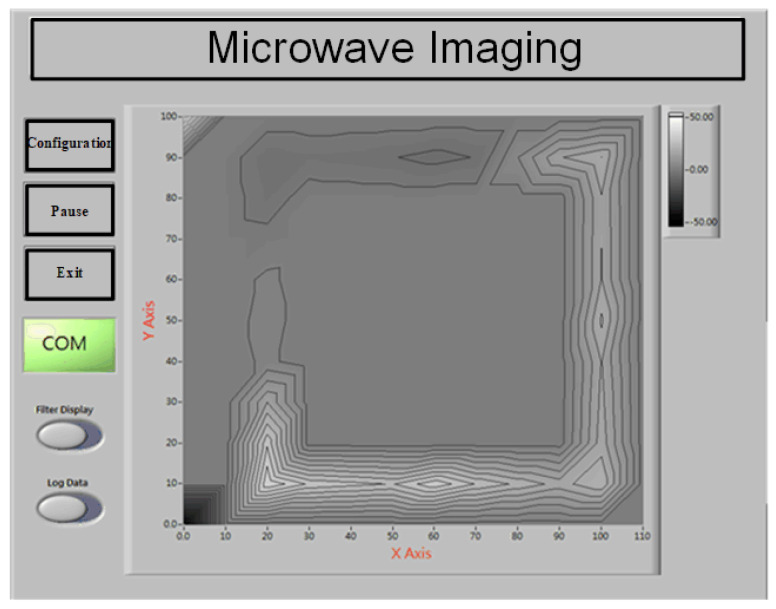
Profile plot of the L-shaped object.

To verify the radar system, two types of experiments were conducted to demonstrate its concept and capabilities. The first experiment involved detecting the object profile at a fixed nominal distance of 15 cm between the antenna module and the object. The sensing distance can be adjusted by delay line control. Due to the low-power nature of the IR-UWB system, short-range detection was employed. The sensing data were observed on a LabVIEW program GUI. In this experiment, a hand was first placed in front of the antenna to observe the variation in the reflected signal, shown in Figure 8. The speed of the switching antenna unit was defined as 0.5 s with a full scan of 4 s. A background data matrix was first measured to obtain the background threshold value, which was in terms of environment. Each testing environment had different antenna threshold values. Subsequently, the radar received the reflected waves based on the switching antennas within a sensing coverage. These waves were further processed by digital signal processing to form a data matrix of the signal strength distribution. Thus, an L-shaped wooden stick was then used to observe the reflected wave based on the signal degradation curve with a 2D profile distribution. Figure 9 depicts a real-time contour plot with great imaging quality on the GUI frame, where the concentrated area represents the object profile based on the proposed algorithm with the iterated τ calculation.

**Figure 10:**
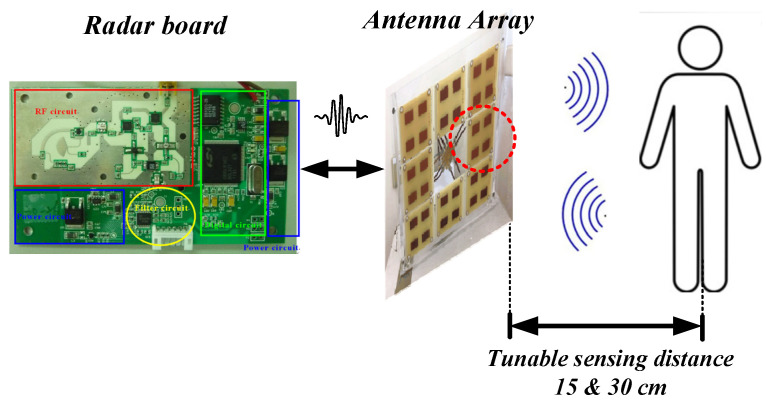
Radar experimental setup for vital sign detection.

**Figure 11:**
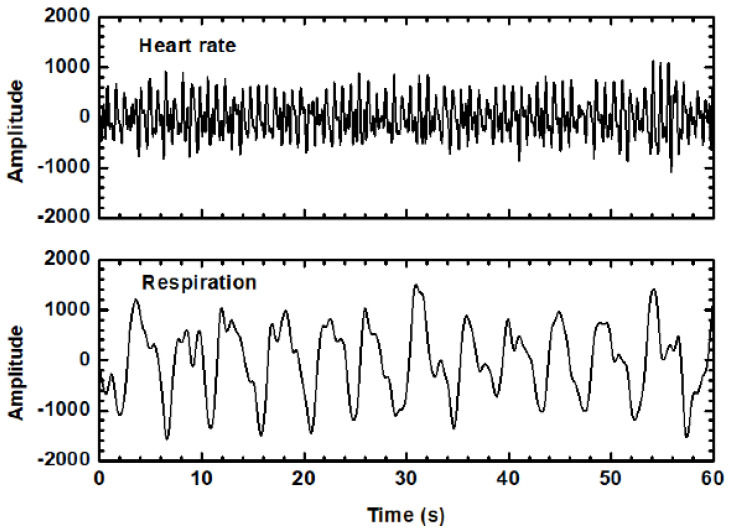
Measured vital sign results from the IR-UWB sensor.

**Figure 12:**
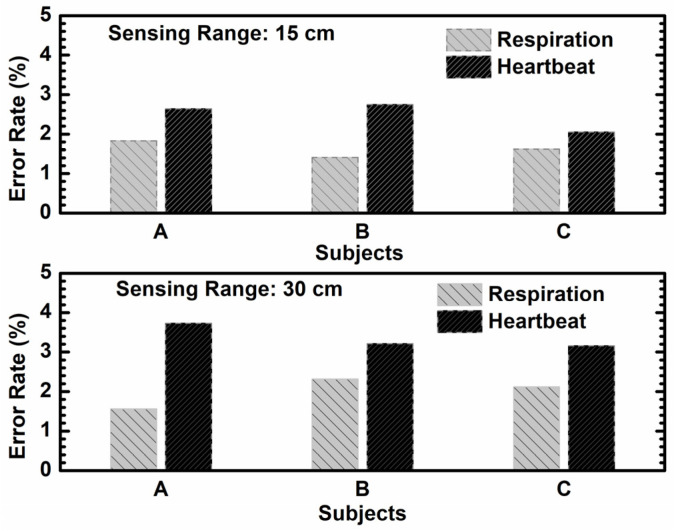
Error rate percentage of vital sign detection with different subjects and distances.

Additionally, to emphasize the effectiveness of the proposed dual-mode embedded IR-UWB radar system in measuring breathing rate and heartbeat, a single antenna unit sensor was first employed for the continuous monitoring of thoracic movements at a fixed distance of 15 cm between the antenna and the subject. Subsequently, a sensing distance of 30 cm was recorded for comparison. Figure 10 illustrates a photograph of the experimental setup to show the actual positions for vital sign detection. The sensing range can be adjusted by the delay time control [23]. However, due to a trade-off between the transmitted power and propagation loss in free space, the maximum detected range is limited to less than 30 cm. The captured vital signs are processed digitally by the MCU using an LPF with a roll-off frequency at 2 Hz. Figure 11 illustrates the recorded time domain vital signs based on radar sensing for a duration of 1 min. The results reveal that the periodical physiological signal behavior can be successfully detected using the proposed sensor. In addition, the reliability of the sensor was evaluated by calculating the error rate percentage, which was determined by comparing the sensor’s breathing and heart rates with reference values. The reference value for respiration was obtained by counting the number of the subject’s breaths during the test, while the reference heartbeat was measured using a commercial product (Guider smart band, 600Z) that utilized a photoplethysmography (PPG) sensing technique. Accordingly, the measured vital sign results can be compared with the reference data to observe the sensing accuracy. Experiments were conducted with three healthy male volunteers (graduate students aged 23 to 25) who were seated directly in front of the radar sensor. The eight sub-array antenna was set to only a single antenna unit for transmitting/receiving vital signs. Measurements were taken at two distinct sensing distances—15 cm and 30 cm—for each participant. Breathing and heartbeat rates were recorded over 30 s to calculate beats per minute and the corresponding percentage error rate. Figure 12 depicts the error rate percentage for vital sign sensing at different distances and subjects. It is noteworthy that the sensor achieved an error rate of less than 2% at a sensing range of 15 cm. As the detection range increased, the sensing accuracy degraded, but the estimated error rates remained below 4%. These results demonstrate the overall reliability of the implemented sensor.

## 4. Conclusions

This study presents an integrated IR-UWB radar system including hardware and algorithm realizations for both microwave imaging detection and vital sign sensing. The ASP was utilized to deal with out-of-band interference while maintaining the DC component for the subsequent self-calibration algorithm for object profile detection. Additionally, an inverse scattering equation with the iterated τ was established to overcome the nonlinear issue. The proposed concept has potential to detect material characteristics along with statistical or artificial intelligent techniques for biomedical applications.

## Figures and Tables

**Table 1:** Specifications of components used in radar board.

Block	Manufacturer	Specifications
RF switch	Analog Devices	DC—8 GHz, 1.2 dB insertion loss
Driving Amplifier	Analog Devices	DC to 6 GHz, 20 dB gain
LNA	Avago	1.5–8 GHz, 23 dB gain, 1.6 dB NF
Downconverter	Analog Devices	4.5–6 GHz, 30 dB LO-RF isolation, 6.5 dB conversion loss
Low Pass Filter	Mini-Circuits	DC—400 MHz, low insertion loss
Low-Dropout Regulator	Texas Instruments	Available in 5 V; Output current: 800 mA
OPA	Analog Devices	1.8–5 V supply and rail to rail
Microcontroller	Texas Instruments	Maximum frequency of 80 MHz
Bluetooth	ITead Studio	HC05 Bluetooth to serial port

## Data Availability

The original contributions presented in the study are included in the article, further inquiries can be directed to the corresponding authors.
